# Quorum sensing inhibition by South African medicinal plants species: an in vitro and an untargeted metabolomics study

**DOI:** 10.1186/s12906-025-04880-4

**Published:** 2025-04-12

**Authors:** Phanankosi Moyo, Olusola Bodede, Madelien Wooding, Ibukun M. Famuyide, Fikile N. Makhubu, Ndivhuwo K. Khorommbi, Michael Ofori, Cynthia A. Danquah, Lyndy J. McGaw, Vinesh J. Maharaj

**Affiliations:** 1 Biodiscovery Center, Department of Chemistry, University of Pretoria, Hatfield, Pretoria, 0028 South Africa; 2 Phytomedicine Programme, Department of Paraclinical Sciences, Faculty of Veterinary Science, University of Pretoria, Private Bag X04, Onderstepoort, Pretoria, 0110 South Africa; 3 Department of Pharmaceutical Sciences, Dr Hilla Limann Technical University, Wa, Ghana; 4 Department of Pharmacology, Faculty of Pharmacy and Pharmaceutical Sciences, College of Health Sciences, Kwame Nkrumah University of Science and Technology, PMB, Kumasi, Ghana

**Keywords:** *Chromobacterium violaceum*, Quorum sensing, Natural products, Plants, Metabolomics, Antimicrobial drug resistance

## Abstract

**Background:**

The emergence of antimicrobial resistance (AMR) is imperiling global health, hence, the need to remedy this challenge by discovering new therapeutic strategies and agents. Quorum sensing inhibition (QSI) is opined as a potential novel strategic approach in the fight against AMR by abrogation of bacterial virulence and pathogenicity. Currently, there are no clinically approved QSI drugs. Based on this, this study evaluated the QSI properties of South African plant species*.*

**Methods:**

Twenty-nine extracts and their corresponding 203 fractions generated using solid phase extraction were screened for QSI activity in vitro against *Chromobacterium violaceum* ATCC 12472. Active and inactive fractions of the most potent plant species were analysed using UPLC-HRMS. The acquired mass spectral data was subjected to chemometric analysis.

**Results:**

From the QSI assays, three plant species showed remarkable QSI activity, measured by dose-dependent inhibition of violacein production (IVP), at sublethal concentrations. *Terminalia phanerophlebia* emerged as the most active species, with the extract and five of its fractions showing good activity in IVP (IVP IC_50_ ≤ 0.1 mg/mL). This was closely followed by *Momordica cardiospermoides* whose crude extract and two of its corresponding fractions showed good activity (IVP IC_50_ ≤ 0.1 mg/mL). Three fractions of *Helichrysum odoratissimum* also had good activity (IVP IC_50_ ≤ 0.1 mg/mL) marking it one of the most potent selected species. Chemometric analysis identified five compounds including olivetol and hydroxytyrosol as chemical markers positively associated with the QSI activity of *T. phanerophlebia*.

**Conclusion:**

In conclusion, the findings of our study provided insight into the QSI properties of South African plant species. Further studies will focus on the isolation of the putative active compounds and the in vitro evaluation of their QSI activity.

**Supplementary Information:**

The online version contains supplementary material available at 10.1186/s12906-025-04880-4.

## Background

Bacterial pathogens have been a pervasive scourge to human species causing a plethora of deadly infectious diseases. Some of the most profoundly grim historical moments of humanity were caused by bacterial pathogens. The most notable in this regard were the “three pandemic plagues” caused by the highly virulent Gram-negative pathogenic bacterium, *Yersinia pestis* [1, 2]. These pandemics caused millions of deaths in a short period of time, changing the very fabric of human society and civilisation [1]. The discovery of antibiotics, i.e., compounds with either bactericidal or bacteriostatic activity, in the twentieth century proved to be a game changer in the fight against bacterial infections [3]. By inhibiting bacterial proliferation and, subsequently, infection, antibiotics allowed the treatment of diseases that previously could not be cured. Although the use of antibiotics for decades resulted in a significant decline in morbidity and mortality associated with bacterial pathogens, the global health burden resulting from infections caused by these microorganisms remains of great proportions [4]. For example, bacterial infections were responsible for 13.6% of global deaths in 2019. Fifty-four percent of these associated bacterial deaths were caused by five bacterial pathogens, namely: Gram-positive *Staphylococcus aureus* and *Streptococcus pneumoniae*; Gram-negative *Escherichia coli*, *Klebsiella pneumoniae* and *Pseudomonas aeruginosa* [4]. Unfortunately, the misuse and abuse of antibiotics has led to the emergence and spread of drug-resistant bacterial phenotypes giving rise to the antibiotic resistance (AMR) phenomenon [5]. Drug-refractory bacterial strains arise due to drug pressure imposed by antibiotics that suppress bacterial growth, resulting in inadvertent selection for mutant bacterial strains that are less susceptible to antibiotics. The challenge of AMR is already having a considerable impact on global health, causing *ca*. 4.5 million fatalities in 2019 [6]. Consequently, there is an urgent need to find alternative approaches to curb bacterial infections. One of such opined strategy is quorum sensing inhibition (QSI) [7–13].

Quorum sensing (QS) is a cell-density-based cell-to-cell bacterial communication system responsible for the coordination and control of a myriad of group bacterial behavioral processes [9, 14, 15]. Bacterial cell-to-cell communication is mediated by small diffusible molecules called auto-inducers (AI). The communication process depends on the production, release, and recognition of AI by receptors. In Gram-negative and Gram-positive bacteria, AI are acyl-homoserine lactones and oligopeptide molecules, respectively. At a specific density of the bacterial population, that is, the quorum, a proportional threshold level of AI signal molecules is attained resulting in their binding to receptors forming a transcription complex [9–11, 14, 15]. The transcription complex drives the expression of specific target genes. Quorum circuit systems coordinate the expression of virulence genes, surface attachment, biofilm formation, and pathogenicity factors in some bacterial species. Virulence genes produce virulence factors that include toxins, virulence enzymes, and adhesion molecules, all of which contribute to the severity of bacterial pathogenicity [9, 10, 14, 15]. Therefore, it is conceivable that by interrupting processes mediated by quorum sensing, it is possible to attenuate bacterial pathogenicity. Quorum sense inhibition can be achieved by targeting the following, (i) AI synthase, i.e., the LuxI-type enzymes that produce AI, (ii) AI itself, e.g., by their degradation, and (iii) the binding of AI to receptors, i.e., the LuxR-type transcriptional activator proteins [9, 14, 15]. The QSI strategy does not suppress bacterial growth and hence does not apply selective pressure on bacteria. This lack of selective pressure implies that the probability of the emergence of resistant bacterial strains to the QSI approach is minimal [16]. This makes QSI an attractive concept, especially considering that some of the most pathogenetic bacteria, including *S. aureus*, enterohemorrhagic *E. coli*, *P. aeruginosa, Vibrio cholera* (a Gram-negative bacterium) and *Klebsiella pneumoniae*, use QS-circuits to control the expression of their virulence genes [17–19].

Owing to its potential, the QSI paradigm has received marked interest in the antimicrobial drug discovery field. The foremost classical exemplary QSI drug that has advanced in clinical trials is azithromycin. This antibiotic, which is not active against *P. aeruginosa,* was shown in a placebo-controlled clinical trial to significantly interfere with QS of this bacterium in intubated patients (Trial registration: ClinicalTrials.gov NCT00610623) [20]. Two clinical trial candidates, sibofimloc and GSK3882347, target a QS-mediated process in *E. coli* [21]. Sibofimloc (TAK- 018) is a first-in-class drug for preventing inflammation associated with Crohn’s disease [22]. This compound is structurally classified as a fluorene by the online chemotaxonomy tool ClassyFire [23]. Sibofimloc works by preventing the surface attachment and biofilm formation of adherent-invasive *E. coli* in patients with Crohn's disease. Adhesion to the intestinal lining by adherent-invasive *E. coli* triggers a chronic immune response synonymous with Crohn’s disease [20]. Sibofimloc is currently undergoing Phase 2 clinical trials (Trial Registration: ClinicalTrials.gov NCT03943446). GSK3882347 has a similar mode of action to sibofimloc in preventing the attachment of *E. coli* albeit in the urinary tract, which consequently eliminates its infection of that organ. GSK3882347 is currently undergoing Phase 1 clinical trials (Trial registration: ClinicalTrials.gov NCT05138822). The QSI pipeline continues to grow with the emergence of more hit, early, late, and pre-clinical candidates [11, 24, 25].

**Fig. 1:**
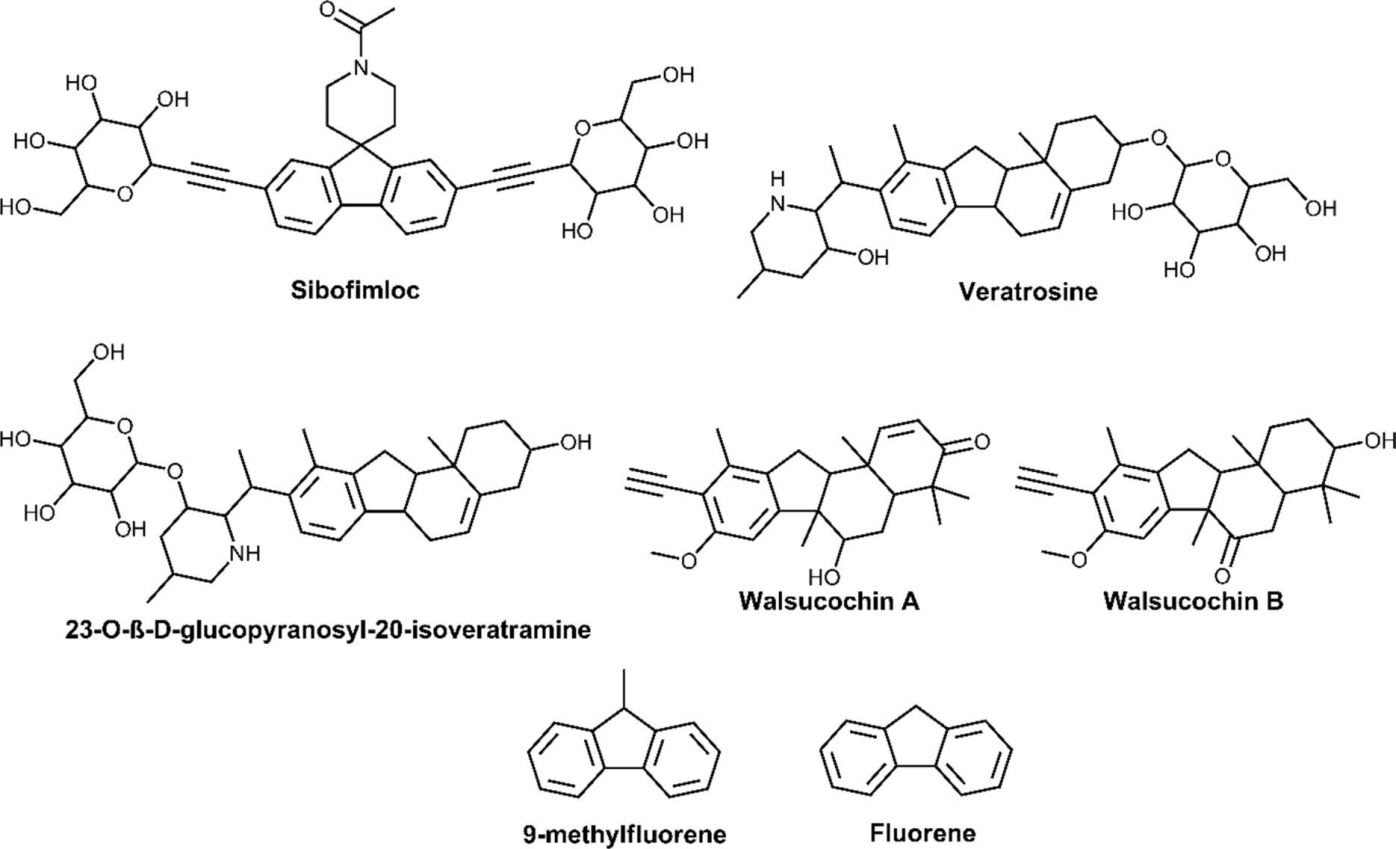
2D chemical representations of sibofimloc and structurally related fluorene compounds found in different plant species. Veratrosine and 23-O-β-D-glucopyranosyl- 20-isoveratramine were isolate from *Veratrum patulum* (Liliaceae) [39]. Walsucochins A and B were Isolated from *Walsura cochinchinensis* (Meliaceae) [40]. 9-methylfluorene and fluorene have been found in *Illicium verum* (Schisandraceae) [41] and *Zea mays* (Poaceae) [42], respectively

One source that has generated great interest in the search for QSI agents is natural products [11, 24, 25]. Investigations on marine and microbial-derived natural products have yielded interesting chemotypes with QSI properties [11, 24, 25]. One of the most interesting classes of compounds identified so far are the furanones [26–28]. Different compounds assigned to this class, including their synthetic derivatives, have been shown to have prolific QSI properties against different pathogens, including *P. aeruginosa*, *Vibrio anguillarum*, *Salmonella enterica*, and *Streptococcus* sp. [11, 24–28]. Plant-derived natural products are also proving to be a potential source for hit and early-to-late lead QSI agents. The most notable are rosmarinic acid, salicylic acid, and vanillin, with the latter compounds routinely used as positive control compounds for QSI assays [11]. Interestingly, rosmarinic acid has been shown to induce premature quorum sensing in *Pseudomonas aeruginosa*. Its secretion by plants is believed to be a defense response mechanism [29]. An interesting feature of plants is that, despite their extensive evaluation in the search for either bactericidal or bacteriostatic compounds, they have not yet yielded a clinically relevant drug. This is even though plants live in environments in which they are exposed to bacterial pathogens. Could they perhaps be using QS inhibiting secondary metabolites to fight infections? And could they finally contribute to the fight against bacterial infections by providing novel scaffolds from which QSI drugs will be developed? Perhaps interesting to note is that plants are a well-documented source of fluorene compounds which are structurally similar to sibofimloc (Fig. 1) [30–38].

In our current study, we assessed the QSI properties of crude plant extracts of South African plant species from 17 families and their solid phase extraction (SPE) generated fractions. We used the model organism *Chromobacterium violaceum* ATCC 12472, a Gram-negative bacterium which produces the purple-coloured pigment violacein upon successfully reaching a quorum [43]. Quorum sensing in *C. violaceum* is regulated by CviI, a LuxI-type homologue, and a transcriptional regulator CviR, a LuxR-type homologue [43–45]. The QSI of *C. violaceum*, at sublethal drug concentrations, results in the failure to produce violacein. Violacein can be clearly seen with the naked eye and additionally can be quantified using spectrophotometric methods, which allows the use of *C. violaceum* as a model biosensor strain to assess samples for QSI activity [43–45]. Using chemometrics analysis, we tentatively identified compounds predicted to be responsible for the observed QSI of the analysed samples.

## Methods

### Plant material collection, extraction, and fractionation

The plant material used in the study was collected from a repository in the Biodiscovery Center, Department of Chemistry, University of Pretoria. The set of plant species studied here consists of those previously collected and prepared by Clarkson et al*.* [46], Fouche et al. [47], Fouche et al. [48], Moyo et al. [49] and Mianda et al*.* [50] (Supplementary File Table S1.1 and S1.2). The plant material investigated included leaves, whole plants, roots, flowers, and mixtures of leaves and flowers. The plant species from the repository were prioritised for the study based on knowledge of their traditional use for the treatment of a plethora of diseases. The influence of seasonality on the activity of the plant extracts and fractions was not considered in the study. We do acknowledge this as a limitation of the investigation that needs to be redressed in future studies.

### Extraction and fractionation

A slightly modified protocol of the methods reported by Invernizzi et al*.* [51] and Thornburg et al*.* [52] was used for the microextraction of dry plant material and their fractionation [51]. A 50 mL mixture of dichloromethane (DCM): methanol (MeOH) (1:1, v/v) was transferred to an extraction vessel containing the plant material (7.2 g). The solvent and plant material mixture was sonicated in an ultrasonic bath for 1 h at a temperature of *ca.* 38ºC [51]. The DCM: MeOH extract was collected into round bottom flasks, before carrying out a second extraction cycle with 50 mL MeOH. Crude extracts were combined and dried using an SP Genevac HT6 (Genevac Ltd., Ipswich, UK). A HypeSep C8 SPE cartridge (2 g/6 ml) was used to fractionate each extract into 7 fractions using a Gilson GX- 241 ASPEC® liquid handler [51]. The crude extract (600 mg) was dissolved in 6 mL of a DCM: MeOH solution and transferred onto a cottonwool roll. The crude extract-impregnated cottonwool roll was dried using an SP Genevac HT6 before being transferred to an empty prewashed 10 mL SPE cartridge [51]. Fractionation was carried out as previously outlined by Invernizzi et al. [51]: 19:1 (H_2_O: MeOH) (Fraction F1); 4:1 (H_2_O: MeOH) (Fraction F2); 3:2 (H_2_O: MeOH) (Fraction F3); 1:1 (H_2_O: MeOH) (Fraction F4); 2:3 (H_2_O: MeOH) (Fraction F5); 1:4 (H_2_O: MeOH) (Fraction F6) and 1:1 (ACN: MeOH) (Fraction F7) resulting in 7 fractions (F1 to F7) collected per each plant crude extract. The collected fractions were dried using an SP Genevac HT6. Dried fractions and extracts were dissolved in 100% dimethyl sulphoxide (DMSO) to a stock solution of 20 mg/mL and stored in a fridge at 4ºC prior to screening.

### Quorum sensing inhibition assay

To assess the ability of extracts and fractions to inhibit QS in vitro in the bioreporter *C. violaceum* ATCC 12472 strain, a slightly modified microdilution method reported by Adeyemo et al*.* [53] was used. *Chromobacterium violaceum* was aerobically cultured in Luria–Bertani (LB) broth overnight at 30 °C in a shaking incubator set at 140 rotations per minute (rpm). A working bacterial suspension was prepared by diluting the overnight culture with LB broth to obtain an absorbance of 0.1 ± 0.02 at a wavelength of 590 nm to match the McFarland standard 0.5 (correlating to 1.5 × 10^8^ CFU/mL).

To perform full-dose response studies, stock solutions (20 mg/mL) of plant extracts and their respective fractions were first diluted in LB broth to 10 mg/mL followed by transfer of 250 µL into two wells on the first column (well 1) on Rows A and B in a sterile 48-well plate which already has 250 µL/well of LB broth in all its wells. Two-fold serial dilutions, using a multichannel pipette (300 µL), across an 8-concentration point range were carried out for both rows in the direction of well 1 to 8 of 48-well plates. The concentration ranges used were 0.019531 to 2.5 mg/mL for most of the extracts and fractions and 0.007813 to 1 mg/mL only for extracts and fractions observed to be highly active in the first independent biological experiment (n = 1). Row A was seeded with an equal volume (250 µL/well) of bacterial suspension (test experiment), while to Row B an equal volume of LB broth (250 µL/well) was added (blank experiment) (total of 500 µL/well). Eight wells in Row C were seeded with bacterial suspension and served as an untreated growth control. Vanillin (at a stock concentration of 10 mg/mL) in Row F served as a positive control for QSI. The plates were sealed with parafilm and incubated at 30 °C overnight (24 h) in a static incubator. Following overnight incubation, the minimal inhibitory concentration (MIC) and minimum quorum sensing inhibitory concentration (MQSIC) were determined. These MIC and MQSIC evaluations were based on bacterial growth and violacein production (a purple pigmentation that forms as an insoluble layer on the air-media surface interphase in wells) as follows: (i) The lowest concentration at which no growth (clear wells) was observed, and no pigmentation was interpreted as MIC. (ii) The lowest concentration with growth (turbid or opaque colour) and no pigmentation was interpreted as MQSIC.

Following determination of both the MIC and MQSIC, 250 µL of culture media was removed from each well in both the test and blank experiments. An equal volume (250 µL) of 100% DMSO was transferred into all wells, forming a 50% solution which, after 10 min of shaking at 140 rpm, completely dissolved the violacein. The plates were then centrifuged for 10 min at 4000 rpm, after which 200 µL/well was transferred to 96-well plates. The amount of violacein in the solution was determined by measuring the absorbance at 595 nm. The ‘background’ absorbance of the blanks, due to extracts and fractions in solution, was subtracted from the total absorbance measured for each test sample, ensuring that the absorbance was exclusively that of violacein in solution. The % inhibition of violacein production (IVP) was calculated using the formula shown below.

$$IVP \left(\%\right)=\frac{\left[Untreated control 595 nm- \left(Test 595 nm - Blank 595 nm \right)\right]}{Untreated control 595 nm } x 100$$

The data obtained were analysed in Microsoft Excel and the sigmoidal dose–response curves were plotted using GraphPad (v5), allowing for the determination of the concentration at which 50% of the IVP occurs. For primary screening, one biologically independent experiment was carried out for all samples. Biological repeats (2 ≤ *n* ≤ 5) were performed for nonlethal samples (those showing ≥ twofold difference (FD) in the MIC and MQSIC values) with a measured IVP IC_50_ ≤ 0.1 mg/mL in primary screens.

#### Ultra-high-performance liquid chromatography (UPLC) Instrumentation and conditions

Ultra-high-performance liquid chromatography (UPLC) analysis of crude extracts and their corresponding SPE generated fractions was carried out using a Waters Acquity UPLC system (Waters Corp., MA, USA). Crude extracts and fractions were dissolved in MeOH (Methanol 215, Super Purity, Romil, Waterbeach, England) to a concentration of 1.5 mg/mL before spinning down for 30 s using a mini centrifuge (Model M- 6, Boeco, Hamburg, Germany). The dissolved crude extracts and fractions were analysed on an ACQUITY UPLC® BEH C18 (2.1 × 100 mm, 1.7 µm) column (Waters Inc., Milford, MA, USA). The gradient method used for separation is provided in Table 1. Solvent system used were (A) H_2_O + 0.1% formic acid (FA) and (B) MeOH + 0.1% FA. Target column temperature was set at 50 °C with a run time of 20 min and an injection volume of 5 μL.

**Table 1:** Gradient table

Time (min)	Flow Rate (mL/min)	%A	%B	Curve
1. Initial	0.30	97.0	3.0	Initial
2. 1.0	0.30	97.0	3.0	6
3. 14.0	0.30	0.0	100.0	6
4. 17.0	0.30	0.0	100.0	6
5. 17.5	0.30	97.0	3.0	6
6. 20.0	0.30	97.0	3.0	6

#### High Resolution Mass Spectrometry (HRMS) instrumentation and conditions

Mass spectral data acquisition was performed on a Waters® Xevo G2-Quadrupole Time-of-Flight (QTOF) mass spectrometry system (Waters Inc., Milford, MA, USA). MassLynx™ (v 4.1) (Waters Inc., Milford, MA, USA) software was used to operate the instrument and for data acquisition. The parameters and conditions were as follows: Polarity – ESI^+^; Mass—50 Da to 1200 Da; Calibration mass range, Start mass (Da)—173.041, End mass (Da)—1071.885; Lock Spray Reference Standard—Leucine Enkephalin, Reference Scan Frequency (sec)—10.000, Lock Spray Infusion Flow Rate (µL/min)—3; Capillary (kV)—2.8000, Source Temperature (°C) – 120, Desolvation Temperature (°C)—350, Cone Gas Flow (L/h)—20.0, Desolvation Gas Flow (L/h)—600.0; Collision Energy (eV)—6.0.

Function Parameters—Function 1—TOF Parent Function (Low energy MS): Survey Start Time (min)—0.0, Survey End Time (min)—20.0; Parent Survey High CE (V)—30.0, Parent Survey Low CE (V)—10.0; Survey Data Format – Continuum; MS Collision Energy (eV)—2.0; Cycle time (sec)—0.314 per Scan, Scan duration (sec)—0.300 (3.3 Scans/sec), Inter Scan Delay (sec)—0.014.

Function Parameters—Function 2—TOF Parent Function (High energy MS): Survey Start Time (min)—0.0, Survey End Time (min)—20.0; Ramp High Energy from 20.0 to 40.0 eV, Parent Survey Low CE (V)—10.0; MS Collision Energy Low (eV)—20.0, MS Collision Energy High (eV)—40.0. Cycle time (sec)—0.314 per Scan, Scan duration (sec)—0.300 (3.3 Scans/sec), Inter Scan Delay (sec)—0.014.

### Data processing, and chemometric analysis

Acquired MS data was processed using MarkerLynx XS Method Editor (v 4.1) (Waters Inc., MA, USA). Parameters used for the processing are outlined in Table 2. Features were generated using a marker tolerance of 0.05 Da for m/z and ± 0.2 min for RT. The data matrix sheet resulting from the pre-processing was exported as a Microsoft Excel comma delimited (.csv) sheet which was used as an input file on SIMCA®-P (v 17) (Sartorius Stedim Data Analytics AB, Umeå, Sweden) for unsupervised principal component analysis (PCA) and supervised Orthogonal Projections to Latent Structures Discriminant Analysis (OPLS-DA) multivariate analysis. The PCA and OPLS-DA models were constructed of Pareto Scale transformed data. For this analysis, the SPE-fractions were categorised into two classes, class 1 (IVP IC_50_ ≤ 0.1 mg/mL) and class 2 (IVP IC_50_ > 0.1 mg/mL). The PCA and OPLS-DA models were visualised as scores and loadings plots. Discriminating variables of significance were noted from the OPLS-DA loadings S-plot, and their corresponding variable importance in the projection (VIP) values (> 1).

**Table 2:** Method parameters for UPLC-HRMS data processing on MarkerLynx XS Method Editor (v 4.1)

Property	Value
Function	1
Analysis type	Peak detection
Initial retention time	0.00
Final retention time	20.00
Low mass	50
High mass	1200
Use relative retention time?	No
Peak width at 5% height (sec)	1.00
Peak-to-peak baseline noise	0.00
Apply smoothing?	No
Marker intensity threshold (counts)	10
Mass window (Da)	0.05
Retention time window (min)	0.20
Noise elimination level	0.00
Deisotope data?	No
Replicate % minimum	0.00

## Results

### Anti-quorum sensing activity of plant extracts and their corresponding SPE fractions

Of the 29 extracts and their respective 203 fractions that were screened, 142 were identified as non-lethal (defined as samples showing ≥ 2-FD in the MIC and MQSIC values) while 90 were identified as lethal (defined as samples showing < 2-FD in the MIC and MQSIC values) (Table 3 and Supplementary File Table S2). The more polar fractions, namely F1, F2, F3 and F4 had the highest number of lethal samples, i.e., 20 (69%), 19 (66%), 14 (48%), and 19 (66%), respectively. Interestingly, the medium polar fractions, namely F5, F6, and F7, had the least number of lethal samples, i.e., 9 (31%), 0 (0%) and 3 (10%), respectively. Among the overly represented families (those with either ≥ 2 species or plant parts investigated), the Asteraceae had the highest representation of lethal samples (67%) closely followed by the Lamiaceae (46%). Meliaceae (21%) and Zingiberaceae (25%) had the lowest numbers of lethal samples. *Terminalia phanerophlebia* (Combretaceae) and *Trichilia emetica* (Meliaceae) had the highest number of non-lethal fractions (7 non-lethal fractions for each species), while *Artemisia afra* (Asteraceae) had the lowest number, with only 1 non-lethal fraction (Table 3).

**Table 3:**
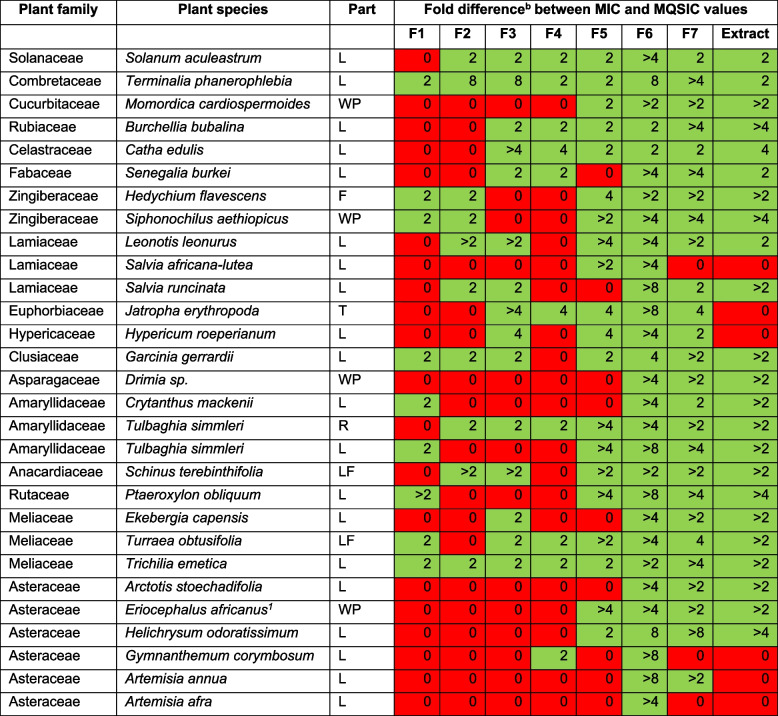
Fold differences between MIC and MQSIC values of plant extracts and fractions

*n* = 1, Red shaded – lethal samples (extracts and fractions, defined as samples showing < 2-FD in MIC and MQSIC values), Green shaded – non-lethal QSI samples (extracts and fractions, defined as samples showing ≥ 2-FD in MIC and MQSIC values). L – leaves, WP – whole plant, R – roots, T – tuber, F – flower, and LF – leaves and flowers.The names and families of plant species were verified in the World Flora Online database (http://www.worldfloraonline.org/)

^a^Eriocephalus africanus var. paniculatus ^2^Fold difference is calculated by dividing the MIC value with the MQSIC value (Supplementary File Table S2)

^b^Fold difference is calculated by dividing the MIC value with the MQSIC value (Supplementary File Table S2)

Following the identification of both lethal and nonlethal extracts and fractions, investigations on the IVP by the nonlethal extracts and fractions commenced. It was important to disregard any samples that showed < twofold difference between the MIC and MQSIC values, as the observed decrease in violacein production could be attributed to the lethal (bactericidal/static) effect of extracts and fractions and not specific QSI mechanisms (Table 4). Of the 142 nonlethal samples, 137 (97%) were shown to have QSI activity (IVP IC_50_ ≤ 1 mg/mL). Twenty-two samples showed good activity QSI (IVP IC_50_ ≤ 0.1 mg/mL) by inhibiting violacein production in a dose-dependent manner (Table 4). Nineteen of the 22 samples were subsequently progressed to more full-dose investigations, generating more data from more biological repeat experiments.

**Table 4:**
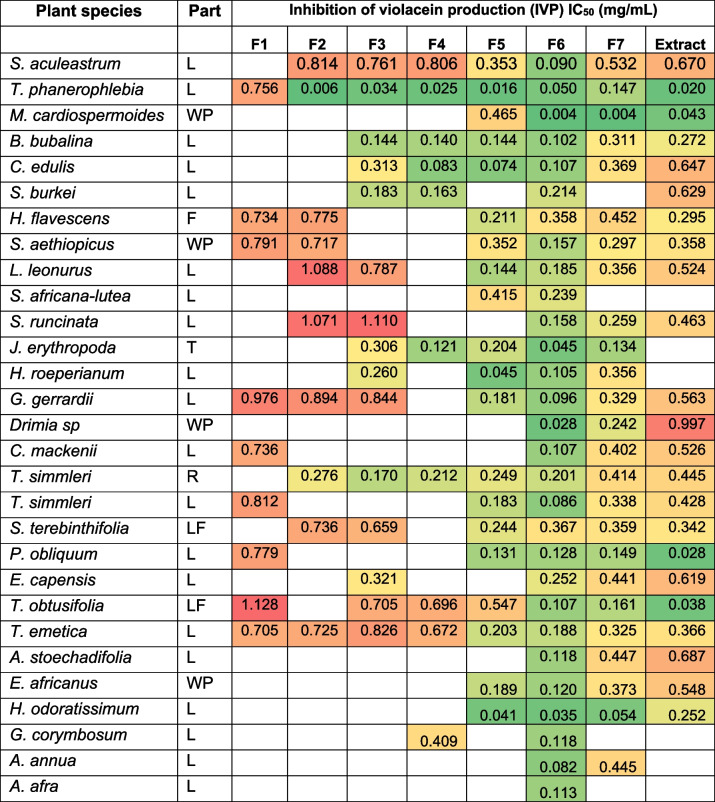
Inhibition of violacein production full-dose investigations

The heat map shows highly active (dark green shaded cells), moderately active (light green-to-yellow-to-light red shaded cells), and inactive (dark red shaded cells)

*n* = 1. L – leaves, WP – whole plant, R – roots, T – tuber, F – flower, and LF – leaves and flowers

*Terminalia phanerophlebia* emerged as the most active QSI plant species. The extract and five of its fractions showed good activity (IVP IC_50_ < 0.1 mg/mL, *n* ≥ 3) with fractions F2 and F4 demonstrating IVP IC_50_ < 0.02 mg/mL (*n* ≥ 3) (Table 5). *Helichrysum odoratissimum* (Asteraceae) and *Momordica cardiospermoides* (Cucurbitaceae) also showed marked potency. Three fractions (F5, 6 and 7) of *H. odoratissimum* showed good activity (IVP IC_50_ ≤ 0.1 mg/mL, *n* ≥ 3) while two of *M. cardiospermoides* also demonstrated this level of activity (IVP IC_50_ ≤ 0.1 mg/mL, *n* ≥ 3) (Table 5). The positive control vanillin had an IVP IC_50_ = 0.101 mg/mL (MIC = 0.625 mg/mL, MQSIC = 0.156 mg/mL, and FD = 4) (*n* = 2) consistent with previous studies [53, 54].

**Table 5:**
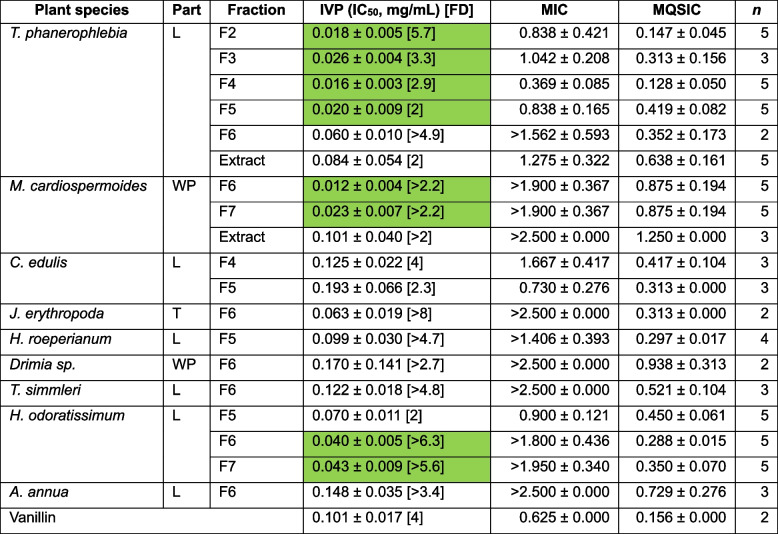
Activity of the most potent fractions in inhibition of violacein production

2≤n≤ 5, Green shaded indicative of IVP IC_50_< 0.05 mg/mL, *FD* Fold difference. Vanillin - (4-Hydroxy- 3-methoxybenzaldehyde)

*MIC* Minimal inhibition concentration and *MQSIC* Minimal quorum sense inhibition concentration, *L* Leaves, *WP* Whole plant, and *T* Tuber

### Chemometric analysis to identify potential QSI chemical markers

**Fig. 2:**
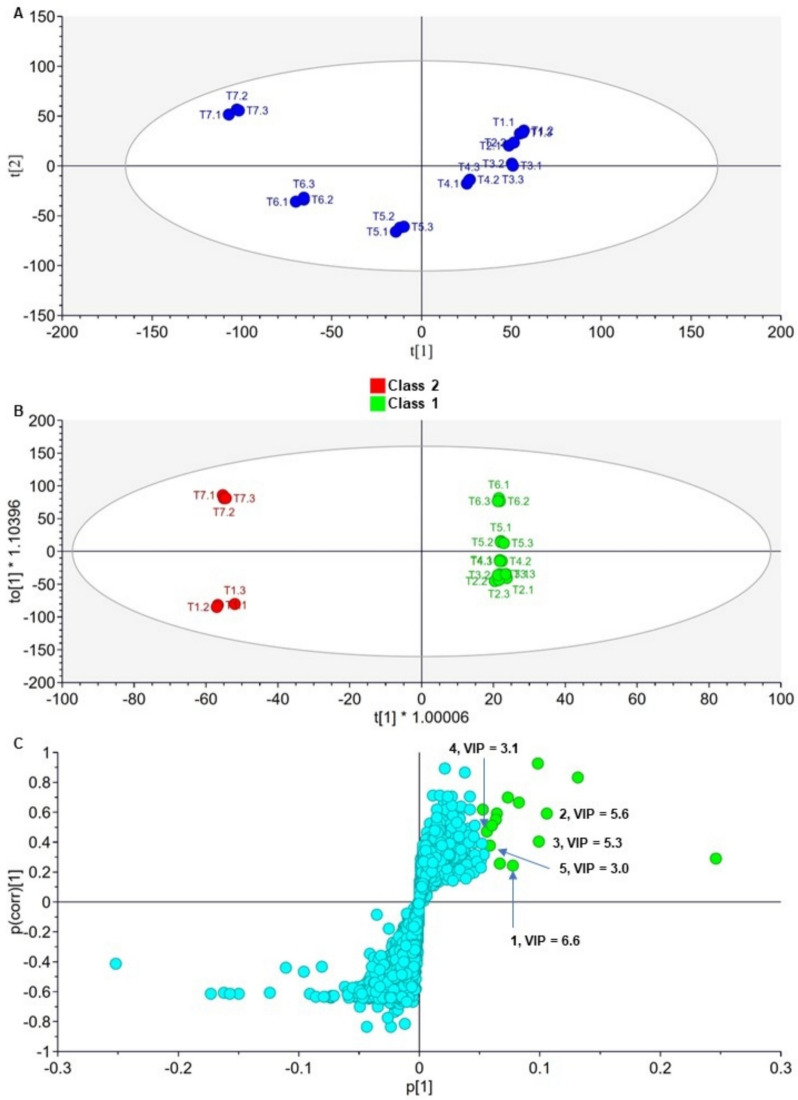
Multivariate analysis of UPLC-MS/MS data of *T. phanerophlebia* fractions using PCA and OPLS-DA models to identify potentially active compounds. Unsupervised (**a**) PCA score plots, (**b**) OPLS-DA score plots, and (**c**) S-plot of OPLS-DA loadings S-plot. OPLS-DA model parameters were as follows; R^2^X = 0.65, R^2^Y = 0.99, and Q^2^ = 0.94. These parameters were indicative that the OPLS-DA model was a good fit with an acceptable predictive capacity. VIP—variable importance in projection. For each fraction, UPLC-HRMS data were acquired in triplicate. The top right quadrant has discriminating features positively associated with the observed pronounced QSI activity of *T. phanerophlebia*. (1) olivetol, (2) hydroxytyrosol, (3) Quercetin- 7-*O*-rhamnoside, (4) vitexin, and (5) 2’’-O-(4-hydroxybenzoyl) hyperin

Having emerged as the most bioactive species, *T. phanerophlebia* was prioritised for chemometric analysis to identify chemical marker compounds positively associated with the observed QSI activity (Fig. 2). Fractions were assigned to two classes based on their QSI activity. The fractions F2 to F6 were classified as having good activity (IVP IC_50_ ≤ 0.1 mg/mL, Class 1) and the fractions F1 and F7 were classified as moderately active (IVP IC_50_ > 0.1 mg/mL, Class 2). The acquired UPLC-HRMS (ESI^+^) mass spectrometry data set for the seven fractions was first pre-processed using Waters MakerLynx XS®. The ESI^+^ mode was used for this analysis as more peaks were detected on it compared to the ESI^−^ mode. The resulting file from the preprocessing step was then used for multivariate analysis on SIMCA®-P.

**Fig. 3:**
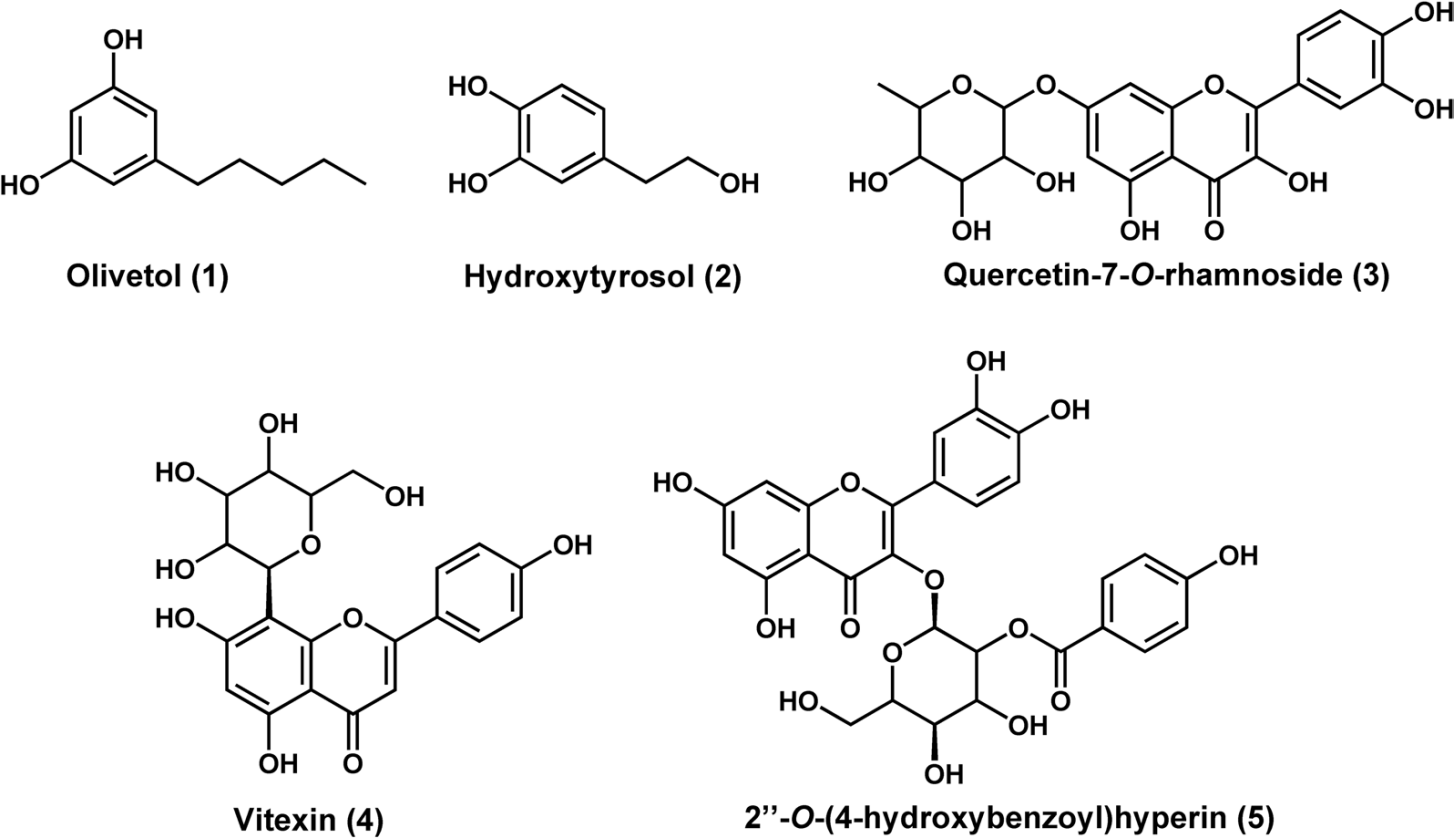
2D chemical representations of tentative compounds from *T. phanerophlebia* predicted to have QSI properties

The principal component analysis (PCA) scores scatter plot showed the different fractions of *T. phanerophlebia* clustering into separate groups (Fig. 2a). Fractions F7, F6 and F5 were distinctly separated from the other clusters. Principal components 1 and 2 accounted for a combined 52.7% of the total variation. Supervised multivariate analysis using Orthogonal Projections to Latent Structures Discriminant Analysis (OPLS-DA) was applied to the UPLC-MS data. The OPLS-DA scores plot clearly showed the separation of the classes 1 (good active) and 2 (moderately active) fractions into two distinct groups along the t1, x-axis (Fig. 2b). To identify biomarker compounds that positively correlate with the observed QSI activity, we generated an S-plot of OPLS-DA loadings (Fig. 2c). The discriminating markers between the two different classes of fractions are observed in the extreme ends of the top right and bottom left quadrants of the loading S-plot. Variables at the extreme end of the top right quadrant, green circles, strongly correlated positively with the observed pronounced QSI activity of the Class 1 fractions (Fig. 2c). To further confirm the positive contribution of these variables, a VIP plot was generated from which VIP scores were provided for all features. All highlighted variables had a VIP score > 1 suggesting that they contributed significantly to marked QSI activity (Fig. 2c). Manual annotation was carried out to tentatively identify the discriminating variables positively associated with the pronounced QSI activity of the Class 1 fractions (Supplementary Files F1 to F5). The Dictionary of Natural Products [55] and published literature sources were used in the manual annotation process [56–60]. The annotation was carried out using the retention time of discriminating markers provided on the S-plots and their corresponding mass-scan (*m/z*) values. This led to the tentative identification of five variables postulated to be active QSI constituents in *T. phanerophlebia*, namely (1) olivetol, (2) hydroxytyrosol, (3) quercetin- 7-*O*-rhamnoside, (4) vitexin, and (5) 2’’-*O*-(4-hydroxybenzoyl)hyperin (Table 6, Fig. 3) [56–60].

**Table 6:** Tentatively identified compounds from *T. phanerophlebia* predicted by the S-plot of OPLS-DA loadings to be active QSI agents

S/N	Rt (min)	Observed mass (m/z)	Calculated mass (m/z)	Mass error (ppm)	Fit Conf %	Adduct	MS/MS fragment Ions (m/z)	Molecular Formula	Compound
1	8.91	203.1029	203.1048	− 9.4^a^	72.2	[M + Na]^+^	181.1258 165.0902 153.0265	C_8_H_16_O_2_	Olivetol
**2**	0.85	177.0521	177.0528	− 4.0	75.7	[M + Na]^+^		C_8_H_10_O_3_	Hydroxytyrosol
**3**	5.99	449.1075	449.1084	− 2.0	84.8	[M + H]^+^	303.0398 165.0297 153.0240	C_21_H_20_O_11_	Quercetin- 7-*O*-rhamnoside
**4**	6.55	433.1127	433.1135	− 1.8	–	[M + H]^+^	415.1000 313.0699 283.0734	C_21_H_20_O_10_	Vitexin
**5**	6.73	585.1241	585.1244	− 0.5	98.6	[M + H]^+^	465.1333 303.0648 153.0214	C_28_H_24_O_14_	2’’-*O*-(4-hydroxybenzoyl)hyperin

*Rt *Retention time, *ppm *parts per million, *Fit conf *Fit confidence. *m/z* – mass to charge

The compounds have been tentatively identified to Confidence Level 3 [61]

^a^Compound annotation outside the < 5 ppm mass error

## Discussion

Our work represents one of the most comprehensive investigations examining the QSI properties of South African plant species. This has led to the identification of three previously undocumented QS-inhibiting plant species, as well as the tentative identification of compounds likely to be responsible for the QSI activity observed for one of the species.

Although some species within the genus *Terminalia* have been studied for their QSI properties [62–66], here we provide the first report on the QSI activity of *T. phanerophlebia.* Compared to other species within this genus, the QSI activity of *T. phanerophlebia* against *C. violaceum* was almost consistent with that of a tannin-rich fraction *Terminalia catappa* that has been reported to inhibit violacein production with a measured IC_50_ value of 0.0625 mg/mL in vitro [63]. Similar to *T. phanerophlebia*, *T. catappa* showed a minimal effect on *C. violaceum growth* with an MIC value of 0.962 mg/mL. *Terminalia bellerica* and *Terminalia macroptera* are other species within this genus that have demonstrated QSI activity against *C. violaceum,* although with less pronounced potency [62, 66]. *Terminalia catappa*, *T. bellerica*, *Terminalia chebula,* and *T. macroptera* have also been observed to attenuate QS against *P. aeruginosa* [62–64]. It may be worth making a similar effort to investigate the QSI activity of *T*. *phanerophlebia* against *P. aeruginosa.*

Chemometric analysis led to the identification of compounds positively associated with the QSI activity of *T. phanerophlebia* against *C. violaceum*. One of the interesting putatively active compounds was tentatively identified as hydroxytyrosol. A Spanish company, Seprox Biotech, successfully filed a patent in 2014 (Publication Number: WO/2014/060581) for hydroxytyrosol and its derivatives as a quorum quenching agent against a broad spectrum of bacterial species involved in food spoilage which also cause food-borne diseases and respiratory tract infections [67]. They were able to validate the QSI of hydroxytyrosol against *C. violaceum,* demonstrating its ability to reduce violacein production in a dose-dependent manner. The structurally related olivetol was also highly correlated with the observed QSI activity of *T. phanerophlebia*. An olivetol rich methanol extract of *Pseudevernia furfuracea* extract was observed to block biofilm formation, a QS-mediated process. The olivetol-rich methanol extract inhibited the formation of biofilms against *S. aureus* (biofilm inhibitory concentration (BIC) = 1.25 mg/mL) and *Proteus mirabilis* (BIC = 0.63 mg/mL) [68]. However, the QSI of olivetol remains to be investigated which will be a subject of future research. The QSI activity of flavonoids has been well established including those of other *Terminalia* species. Most studies have demonstrated the ability of flavonoids to disrupt processes such as biofilm formation. This could substantiate the findings of this study in which three flavonoids are positively associated with the observed QSI activity of *T. phanerophlebia* against *C. violaceum*. While there is a paucity of knowledge on the QSI activity of vitexin against *C. violaceum*, a sub-MIC of vitexin was shown to attenuate QS and biofilm formation in *P. aeruginosa*. Likewise, there is limited information on the QSI activity of quercetin- 7-*O*-rhamnoside against any bacterial species. However, structurally related compounds, including quercetin, have been shown to have a pronounced QSI against different bacteria, including *C. violaceum* and *P. aeruginosa*. The isolation and evaluation of these flavonoids will be a subject of future research.

*Helichrysum odoratissimum*, commonly known by its vernacular name'imphepo', is one of the most prominent medicinal plant species in South Africa. While its antibacterial activity has been extensively investigated [69–72], comparatively, minimal effort has been made to investigate its ability to abrogate QS-circuit controlled processes. However, in their study, De Canha et al*.* [73] showed that a methanolic extract of *H. odoratissimum* has antivirulence activity against the Gram-positive bacterium *Cutibacterium acnes*. While it demonstrated exceptional bactericidal activity against *C. acnes* with an MIC value of 0.00781 mg/mL, the methanolic extract of *H. odoratissimum* prevented bacterial adhesion and disrupted biofilms at a sub-lethal concentration [73]. *Momordica cardiospermoides* has received little attention within the natural product drug discovery field. A PubMed query made on the 12 th of January 2024 using the term “*Momordica cardiospermoides*” only retrieved two manuscripts on this plant species. There is a huge knowledge gap in this species, which we have shown holds great promise as a potential source of QSI agents.

## Conclusions

This study has provided an interesting insight into the QSI properties of selected South African medicinal plant species. We have identified three particularly interesting species, with *T. phanerophlebia*, and *H. odoratissimum* emerging as the most promising. Chemometric analysis led to the identification of five tentatively identified compounds that were hypothesised to be responsible for the QSI properties of *T. phanerophlebia, the* most notably olivetol and hydroxytyrosol. Future studies will focus on the isolation and evaluation of these compounds for their QSI activity, not only against *C. violaceum* but also against other Gram-negative clinically relevant bacterial species including *P. aeruginosa.*

## Supplementary Information

Supplementary Material 1

## Data Availability

The datasets used and/or analyzed during the current study are available from the corresponding author on reasonable request.

## Abbreviations

AMR: Antimicrobial resistance
UPLC-HRMS: Ultra-Performance Liquid Chromatography-High-Resolution Mass Spectrometry
MIC: Minimal inhibitory concentration
MQSIC: Minimum quorum sensing inhibitory concentration
QSI: Quorum sensing inhibition
PCA: Principal Component Analysis
IVP: Inhibition of violacein production
OPLS-DA: Orthogonal Projections to Latent Structures Discriminant Analysis
