# Supplementary material for: Quorum sensing inhibition by South African medicinal plants species: an in vitro and an untargeted metabolomics study
Source: BMC Complement Med Ther. 2025 Apr 12;25:138. doi: 10.1186/s12906-025-04880-4 (PMC11994000; doi:10.1186/s12906-025-04880-4)
Supplement: Supplementary file 1 — Supplementary Material 1 [file 12906_2025_4880_MOESM1_ESM.docx]

Supporting Information

**Quorum sensing inhibition by South African medicinal plants species: An *in vitro* and an untargeted metabolomics study**

Phanankosi Moyo^1^, Olusola Bodede^1^, Madelien Wooding^1^, Ibukun M. Famuyide^2^, Fikile N. Makhubu^2^, Ndivhuwo K. Khorommbi^1^, Michael Ofori^3,4^, Cynthia A. Danquah^4^, Lyndy J. McGaw^2,^*, and Vinesh J. Maharaj^1,^*

^1^ Biodiscovery Center, Department of Chemistry, University of Pretoria, Hatfield, Pretoria 0028, South Africa.

^2^Phytomedicine Programme, Department of Paraclinical Sciences, Faculty of Veterinary Science, University of Pretoria, Private Bag X04, Onderstepoort, Pretoria 0110, South Africa.

^3^Department of pharmaceutical sciences, Dr Hilla Limann Technical University, Wa Ghana

^4^Department of Pharmacology, Faculty of Pharmacy and Pharmaceutical Sciences, College of Health Sciences, Kwame Nkrumah University of Science and Technology, PMB, Kumasi Ghana.

Phanankosi Moyo ([phanankosimoyo@gmail.com](mailto:phanankosimoyo@gmail.com))

Olusola Bodede ([olusolabodede@gmail.com](mailto:olusolabodede@gmail.com))

Madelien Wooding ([madelien.wooding@up.ac.za](mailto:madelien.wooding@up.ac.za))

Ibukun M. Famuyide ([adeyerimi@gmail.com](mailto:adeyerimi@gmail.com))

Fikile N. Makhubu ([fnmakhubu@gmail.com](mailto:fnmakhubu@gmail.com))

Ndivhuwo K. Khorommbi ([khorombink@gmail.com](mailto:khorombink@gmail.com))

Michael Ofori ([michof2825@gmail.com](mailto:michof2825@gmail.com))

Cynthia A. Danquah ([cadanquah.pharm@knust.edu.gh](mailto:cadanquah.pharm@knust.edu.gh))

Lyndy J. McGaw ([lyndy.mcgaw@up.ac.za](mailto:lyndy.mcgaw@up.ac.za))

Vinesh J. Maharaj ([vinesh.maharaj@up.ac.za](mailto:vinesh.maharaj@up.ac.za))

*Corresponding author: Vinesh J. Maharaj (Natural Products Chemistry Analysis)

Tel: +27 (0824665466)

Email address: vinesh.maharaj@up.ac.za

Biodiscovery Center

Department of Chemistry

University of Pretoria

Private Bag x 20

Hatfield, 0028

South Africa

*Corresponding author: L. J. McGaw (Anti-quorum Sensing Analysis)

Tel: +27 (0)12 529 8351

Email address: [lyndy.mcgaw@up.ac.za](mailto:lyndy.mcgaw@up.ac.za)

Phytomedicine Programme

Department of Paraclinical Sciences

Faculty of Veterinary Science

University of Pretoria

Private Bag X04

Onderstepoort

Pretoria 0110

South Africa

Table S1.1: Plants species selected for *in vitro* antiquorum sensing activity screening.

| **Plant species (Family)** | **Voucher number (Herbarium)** | **Part** | **Local name*** | **Traditional use*** |
| --- | --- | --- | --- | --- |
| *Solanum aculeastrum* Dunal. (Solanaceae) | DS03080 | L | Murulwa (Venda), Umthuma, itunga (Xhosa) and thola (Tswana). | Used for the treatment of different human and animal ailments [1]. |
| *Terminalia phanerophlebia* Engl. & Diels. (Combretaceae) | JM00243 | L | AmaNgwe-amnyama, amaNgwe-omphofu (Zulu) and mambonjwane (Swati). | Used for the treatment of many diseases including pneumonia [1]. |
| *Momordica cardiospermoides* Klotzsch. (Cucurbitaceae) | HV00555 | WP | Inshubaba (Swati) and ntwe (Tswana). | Used traditionally for the treatment of different diseases [2, 3]. |
| *Burchellia bubalina* (L.f.) Sims. (Rubiaceae) | BP00936 | L | Thobankomo (Xhosa), Golwane (Zulu) and Hlosana (Swati). | The plant is widely used in folk medicine [4-6] and ethnoveterinary including for the treatment of heart water [7]. |
| *Catha edulis* (Vahl) Endl. (Celastraceae) | PRU 121392 | L | Umhlwazi (Zulu), iqgwaka (Xhosa), lehlatse, lewang, and molomomonate (Sepedi) [8]. | It is used for the treatment of common cold and respiratory of disorders [9] including tuberculosis [8] amongst others. |
| *Senegalia burkei* (Benth.) Kyal. & Boatwr. (Fabaceae) | JM00311 | L | Umkhaya wehlalahlathi, umbabampala (Zulu), umkhaya (Swati), mokgwa (Tswana), and munanga (Venda). | Used to treat eye and back pain [10]. |
| *Hedychium flavescens* Carey ex Roscoe. (Zingiberaceae) | DS02802 | S | Wild ginger (English) [11]. | Used for ritual purposes generally and additionally for traditional medicinal use [11, 12]. |
|  |  | F |  |  |
| *Siphonochilus aethiopicus* (Schweinf.) B.L.Burtt. (Zingiberaceae) | PRE 34817 | WP | Indungulo, isiphephetho (Zulu) and African ginger (English) [13]. | Used for the treatment of many disorders including tuberculosis [8], malaria, asthma, and inflammation [13, 14]. |
| *Leonotis leonurus* (L.) R.Br.  (Lamiaceae) | PRU 121393 | L | Wilde dagga (Afrikaans), umfincafincane, (isiXhosa), and utshwala-bezinyoni (isiZulu). | Its widely used in traditional medicine including for influenza, epilespsy, chest infection, tuberculosis and headaches [8, 15]. |
| *Salvia africana-lutea* L. (Lamiaceae) | FP00851 | L | Ssand sage (English), bruinsalie, sandsalie, strandsalie, and geelblomsalie (Afrikaans). | Used for the treatment of tuberculosis, influenza, common cold fever amongst many other ailments [16]. |
| *Salvia runcinata* L.f. (Lamiaceae) | JS00182 | L | Isicakathi [1]. | The plant is used as a disinfectant and for the treatment of hives [17], burns and sores [18]. |
| *Jatropha erythropoda* Pax & K.Hoffm. (Euphorbiaceae) | HV00572 | T | Rooikambroo (Afrikaans)^1^ and Thotamadi (Tswana and Sepedi) [19, 20]. | Plant is administered as an immune booster for HIV/AIDS management [19] and other sexual transmitted diseases [20]. |
| *Hypericum roeperianum* G.W.Schimp. ex A.Rich. (Hypericaceae) | MM00136 | L | Isivumelelwane (Zulu) [1]. | Used for treatment of different disorders including diarrhoea, pain, indigestion and bacterial diseases [21]. |
| *Garcinia gerrardii* Harv. ex Sim.  (Hypericaceae) | DS02273 | L | Bosgeelmelkhout (Afrikaans), umbande (Xhosa), isibinda, (Zulu) and sikhwelamkhala (Swati). | Used traditionally for the treatment of tuberculosis [8]. |
| *Drimia sp.* (Asparagaceae) | FP00938 | WP |  | Related species used for tuberculosis treatment [8]. |
| *Cyrtanthus mackenii* Hook.f*.*  (Amaryllidaceae) | DS04216 | L | Ifafa lily (English), and impingizana encane empofu (Zulu) [1]. | Used for treatment of cough, headache, cystitis and leprosy [22]. |

*Data collated from PlantZAfrica (<https://pza.sanbi.org/about>) which is provided by the South African National Biodiversity Institute (<https://www.sanbi.org/>). Additional information on plants has been acquired from other sources including published peer reviewed articles. Traditional uses provided are not exhaustive. ^1^<http://redlist.sanbi.org/species.php?species=576-6>. ^2^<https://www.inaturalist.org/> and <https://invasives.org.za/fact-sheet/brazilian-pepper-tree/> (Retrieved on the 1^st^ of April 2024). ^3^<https://www.herbgarden.co.za/mountainherb/herbinfo.php?id=215>.

Continued Table S1.1: Plants species selected for *in vitro* antiquorum sensing activity screening.

| **Plant species (Family)** | **Voucher number** | **Part** | **Local name*** | **Traditional use*** |
| --- | --- | --- | --- | --- |
| *Tulbaghia simmleri* Beauverd (Amaryllidaceae) | FP01384 | R  L | Sweet wild garlic, sweet garlic, fragrant tulbaghia (English), and soetwildeknoffel (Afrikaans). | Used for ornamental purposes. Related species used for tuberculosis treatment [8]. |
| *Schinus terebinthifolia* Raddi (Anacardiaceae) | BP00904 | LF | Brazilian pepper tree (English) and Brasiliaanse peperboom (Afrikaans)^2^. | Used for the treatment of many disorders including gonorrhoea, tuberculosis, and bronchitis^2^. |
| *Ptaeroxylon obliquum* (Thunb.) Radlk.  (Rutaceae) | DS02499 | L | Mogabaletswana (Sepedi) [8] and umThathi (Xhosa). | Used traditionally for the treatment of tuberculosis [8], fever, arthritis and rheumatism [23]. |
| *Ekebergia capensis* Sparrm. (Meliaceae) | FP00739 | L | Cape ash, dogplum (English), essenhout (Afrikaans), umnyamatsi (Swati), nyamaru (Tswana). | Used to facilitate child birth, treat headaches, and skin diseases [24]. |
| *Turraea obtusifolia* Hochst. (Meliaceae) | PRU 129518 | LF | Dima (Sepedi) [19]. | Used traditionally for the treatment of tuberculosis [8]. |
| *Trichilia emetica* Vahl (Meliaceae) | PRU121390 | L | Umkhuhlu (Xhosa), nkulu (Tsonga) and mutuhu (Venda) | Traditional uses include dermatitis, digestive infections, eye infection, malaria and pneumonia [25] |
| *Arctotis stoechadifolia* P.J.Bergius  (Asteraceae) | FP00817 | L | Trailing arctotis (English), kusgousblom, bittergousblom, and witgousblom (Afrikaans). | Used to “clean kidneys” [26]. |
| *Eriocephalus africanus* var*. paniculatus* (Cass.) M.A.N.Müll., P.P.J.Herman & Kolberg (Asteraceae) | FP00896 | WP | Wild rosemary (English), wilderoosmaryn, and kapokbos (Afrikaans). | Used for many diseases including coughs, cold, expectorant, and antimicrobial agent [27]. |
| *Helichrysum odoratissimum* (L.) Sweet. (Asteraceae) | FP01629 | L | Kooigoed, kruie (Afrikaans) and imphepho (Xhosa and Zulu). | Used as an incense, urinary tract infections, fever [1, 28]. Related species used for tuberculosis treatment [8]. |
| *Gymnanthemum corymbosum* (Thunb.) H.Rob. (Asteraceae) | HV00485 | L | Mountain vernonia (English) and uhlunguhlungu (Zulu). | Used to treat fever, and malaria [29]. |
| *Artemisia annua* L. (Asteraceae) (Cultivated) |  | L | Sweet wormwood (English)^3^. | Used for the treatment of fever, and malaria [30]. |
| *Artemisia afra* Jacq. ex Willd.  (Asteraceae) | PRU 121389 | L | African wormwood (English), wilde-als (Afrikaans), mhlonyane (isiZulu) and lengana (Tswana). | Used for tuberculosis treatment [8, 26] amongst a plethora of ailments [31]. |

Table S1.2: Plants species selected for *in vitro* antiquorum sensing activity screening.

| **Plant species** | **Herbarium** | **Name of collectors** | **Collection Site GPS Coordinates** |
| --- | --- | --- | --- |
| *S. aculeastrum* | South African National Biodiversity Institute, National Herbarium, South Africa | Schuhardt, D | 31,37,642S;29,29,779E |
| *T. phanerophlebia* | South African National Biodiversity Institute, National Herbarium, South Africa | Male, JM | 24,31,994S;30,47,316E |
| *M. cardiospermoides* | South African National Biodiversity Institute, National Herbarium, South Africa | Vahrmeijer, H | 27,24,82S;32,6,39E |
| *B. bubalina* | South African National Biodiversity Institute, National Herbarium, South Africa | SANBI* | 28,57,455S;31,45,592E |
| *C. edulis* | H.G.W.J. Schweickerdt Herbarium of the University of Pretoria, South Africa | Moyo, P, Awandu SS, and Andayi, A | -25.625S;28.125E |
| *S. burkei* | South African National Biodiversity Institute, National Herbarium, South Africa | Male, JM | 24,48,64S;30,48,263E |
| *H. flavescens* | South African National Biodiversity Institute, National Herbarium, South Africa | Schuhardt, D | 31,36,304S;29,28,584E |
| *S. aethiopicus* | South African National Biodiversity Institute, National Herbarium, South Africa | Van Warmelo, NJ | 23°0′0″S 29°52′0″E |
| *L. leonurus* | H.G.W.J. Schweickerdt Herbarium of the University of Pretoria, South Africa | Moyo, P, Awandu SS, and Andayi, A | -25.625S;28.125E |
| *S. africana-lutea* | South African National Biodiversity Institute, National Herbarium, South Africa | Potgieter, FJ | 33,58,202S;25,13,359E |
| *S. runcinata* | South African National Biodiversity Institute, National Herbarium, South Africa | Spies, JD | 28,41,291S;28,18,88E |
| *J. erythropoda* | South African National Biodiversity Institute, National Herbarium, South Africa | Vahrmeijer, H | 26,49,44S;20,38,41E |
| *H. roeperianum* | South African National Biodiversity Institute, National Herbarium, South Africa | McMahon, M and Spies, C | 24,49,299S;30,49,450E |
| *G. gerrardii* | South African National Biodiversity Institute, National Herbarium, South Africa | Schuhardt, D | 31,35,868S;29,32,8E |
| *Drimia sp.* | South African National Biodiversity Institute, National Herbarium, South Africa | Potgieter, FJ | 33,56,699S;25,19,425E |
| *C. mackenii* | South African National Biodiversity Institute, National Herbarium, South Africa | Schuhardt, D | 31,35,290S;29,31,4E |
| *T. simmleri* | South African National Biodiversity Institute, National Herbarium, South Africa | Potgieter, FJ | 33,58,208S;25,36,195E |
| *S. terebinthifolia* | South African National Biodiversity Institute, National Herbarium, South Africa | SANBI* | 29,31,306S;31,12,82E |
| *P. obliquum* | South African National Biodiversity Institute, National Herbarium, South Africa | Schuhardt, D | 31,35,990S;29,29,717E |
| *E. capensis* | South African National Biodiversity Institute, National Herbarium, South Africa | Potgieter, FJ | 33,54,311S;25,13,43E |
| *T. obtusifolia* | H.G.W.J. Schweickerdt Herbarium of the University of Pretoria, South Africa | Moyo, P, Awandu SS, and Andayi, A | -25.625S;28.125E |
| *T. emetica* | H.G.W.J. Schweickerdt Herbarium of the University of Pretoria, South Africa | Moyo, P, Awandu SS, and Andayi, A | -25.625S;28.125E |
| *A. stoechadifolia* | South African National Biodiversity Institute, National Herbarium, South Africa | Potgieter, FJ | 32,14,5S;24,32,5E |
| *E. africanus* | South African National Biodiversity Institute, National Herbarium, South Africa | Potgieter, FJ | 33,58,125S;25,13,452E |
| *H. odoratissimum* | South African National Biodiversity Institute, National Herbarium, South Africa | Potgieter, FJ | 33,58,716S;24,14,920E |
| *G. corymbosum* | South African National Biodiversity Institute, National Herbarium, South Africa | Vahrmeijer, H | 27,22,46S;31,59,72E |
| *A. afra* | H.G.W.J. Schweickerdt Herbarium of the University of Pretoria, South Africa | Moyo, P, Awandu SS, and Andayi, A | -25.625S;28.125E |

*SANBI – the collectors where staff members of the South African National Biodiversity Institute. Their names have not been specified. Plant material collected and deposited at the H.G.W.J. Schweickerdt Herbarium of the University of Pretoria, South Africa was identified by Mr. J. Sampson Sampson (Curator at Manie van der Schijff Botanical Garden at the University of Pretoria).

Table S2: Antimicrobial activity of crude plant extracts and their corresponding solid phase generated fractions against *C. violaceum*.

| Plant species | Part | MIC and MQSIC values (mg/mL) | | | | | | | | | | | | | | | |
| --- | --- | --- | --- | --- | --- | --- | --- | --- | --- | --- | --- | --- | --- | --- | --- | --- | --- |
|  |  | F1 | | F2 | | F3 | | F4 | | F5 | | F6 | | F7 | | Extract | |
|  |  | **MIC** | **MQSIC** | **MIC** | **MQSIC** | **MIC** | **MQSIC** | **MIC** | **MQSIC** | **MIC** | **MQSIC** | **MIC** | **MQSIC** | **MIC** | **MQSIC** | **MIC** | **MQSIC** |
| *S. aculeastrum* | L | 2.50 | 2.50 | 2.50 | 1.25 | 2.50 | 1.25 | 2.50 | 1.25 | 2.50 | 1.25 | >2.50 | 0.63 | 2.50 | 1.25 | 2.5 | 1.25 |
| *T. phanerophlebia* | L | 2.50 | 1.25 | 0.63 | 0.078 | 1.25 | 0.16 | 0.31 | 0.16 | 0.63 | 0.31 | 0.63 | 0.078 | >2.50 | 0.63 | 1.25 | 0.63 |
| *M. cardiospermoides* | WP | 2.50 | 2.50 | 2.50 | 2.50 | 2.50 | 2.50 | 1.25 | 1.25 | 2.50 | 1.25 | >2.50 | 1.25 | >2.50 | 1.25 | >2.50 | 1.25 |
| *B. bubalina* | L | 1.25 | 1.25 | 1.25 | 1.25 | 0.63 | 0.31 | 1.25 | 0.63 | 1.25 | 0.63 | 0.63 | 0.31 | >2.50 | 0.63 | >2.50 | 0.63 |
| *C. edulis* | L | 1.25 | 1.25 | 1.25 | 1.25 | >2.50 | 0.63 | 1.25 | 0.31 | 0.63 | 0.31 | 0.63 | 0.31 | 0.63 | 0.31 | 1.25 | 0.31 |
| *S. burkei* | L | 2.50 | 2.50 | 1.25 | 1.25 | 0.63 | 0.31 | 1.25 | 0.63 | 0.63 | 0.63 | >2.50 | 0.63 | >2.5 | 0.63 | 1.25 | 1.25 |
| *H. flavescens* | F | 2.50 | 1.25 | 2.50 | 1.25 | 1.25 | 1.25 | 1.25 | 1.25 | 2.50 | 0.63 | >2.50 | 1.25 | >2.5 | 1.25 | >2.5 | 1.25 |
| *S. aethiopicus* | WP | 2.50 | 1.25 | 2.50 | 1.25 | 1.25 | 1.25 | 1.25 | 1.25 | >2.50 | 1.25 | >2.50 | 0.63 | >2.50 | 0.63 | >2.50 | 0.63 |
| *L. leonurus* | L | 1.25 | 1.25 | >2.50 | 1.25 | >2.50 | 1.25 | 1.25 | 1.25 | >2.50 | 0.63 | >2.50 | 0.63 | >2.5 | 1.25 | 2.5 | 1.25 |
| *S. africana-lutea* | L | 2.50 | 2.50 | 2.50 | 2.50 | 2.50 | 2.50 | 2.50 | 2.50 | >2.50 | 1.25 | >2.50 | 0.63 | 1.25 | 1.25 | 1.25 | 1.25 |
| *S. runcinata* | L | 2.50 | 2.50 | 2.50 | 1.25 | 2.50 | 1.25 | 2.50 | 2.50 | 1.25 | 1.25 | >2.50 | 0.31 | 1.25 | 0.63 | >2.50 | 1.25 |
| *J. erythropoda* | T | 1.25 | 1.25 | 1.25 | 1.25 | >2.50 | 0.63 | 2.50 | 0.63 | 2.50 | 0.63 | >2.50 | 0.31 | 2.50 | 0.63 | 1.25 | 1.25 |
| *H. roeperianum* | L | 1.25 | 1.25 | 1.25 | 1.25 | 2.50 | 0.63 | 0.63 | 0.63 | 1.25 | 0.31 | >2.5 | 0.63 | 1.25 | 0.63 | 1.25 | 1.25 |
| *G. gerrardii* | L | 2.50 | 1.25 | 2.50 | 1.25 | 2.50 | 1.25 | 2.50 | 2.50 | 1.25 | 0.63 | 2.50 | 0.63 | >2.50 | 1.25 | >2.50 | 1.25 |
| *Drimia sp* | WP | 1.25 | 1.25 | 1.25 | 1.25 | 1.25 | 1.25 | 1.25 | 1.25 | 1.25 | 1.25 | >2.50 | 0.63 | >2.50 | 1.25 | >2.50 | 1.25 |
| *C. maackenii* | L | 2.50 | 1.25 | 2.50 | 2.50 | 1.25 | 1.25 | 1.25 | 1.25 | 1.25 | 1.25 | >2.50 | 0.63 | 2.50 | 1.25 | >2.50 | 1.25 |
| *T. simmleri* | R | 1.25 | 1.25 | 1.25 | 0.63 | 0.63 | 0.31 | 0.63 | 0.31 | >2.50 | 0.63 | >2.50 | 0.63 | >2.50 | 1.25 | >2.50 | 1.25 |
| *T. simmleri* | L | 2.50 | 1.25 | 1.25 | 1.25 | 1.25 | 1.25 | 2.50 | 2.50 | >2.50 | 0.63 | >2.50 | 0.31 | >2.5 | 0.63 | >2.5 | 1.25 |
| *S.* *terebinthifolia* | LF | 2.50 | 2.50 | >2.50 | 1.25 | >2.50 | 1.25 | 1.25 | 1.25 | >2.50 | 1.25 | >2.50 | 1.25 | >2.50 | 1.25 | >2.50 | 1.25 |
| *P. obliquum* | L | 2.50 | 1.25 | 2.50 | 2.50 | 2.50 | 2.50 | 2.50 | 2.50 | >2.50 | 0.63 | >2.50 | 0.31 | >2.50 | 0.63 | >2.50 | 0.63 |
| *E. capensis* | L | 2.50 | 2.50 | 2.50 | 2.50 | 2.50 | 1.25 | 0.63 | 0.63 | 1.25 | 1.25 | >2.50 | 0.63 | .>2.50 | 1.25 | >2.50 | 1.25 |
| *T. obtusifolia* | LF | 2.50 | 1.25 | 2.50 | 2.50 | 2.50 | 1.25 | 2.50 | 1.25 | >2.50 | 1.25 | >2.50 | 0.63 | 2.50 | 0.63 | >2.50 | 1.25 |
| *T. emetica* | L | 2.50 | 1.25 | 2.50 | 1.25 | 2.50 | 1.25 | 2.50 | 1.25 | 2.50 | 1.25 | >2.50 | 1.25 | >2.50 | 0.63 | >2.50 | 1.25 |
| *A. stoechadifolia* | L | 2.50 | 2.50 | 2.50 | 2.50 | 2.50 | 2.50 | 1.25 | 1.25 | 1.25 | 1.25 | >2.50 | 0.63 | >2.50 | 1.25 | >2.50 | 1.25 |
| *E. africanus* | WP | 1.25 | 1.25 | 1.25 | 1.25 | 1.25 | 1.25 | 1.25 | 1.25 | >2.50 | 0.63 | >2.50 | 0.63 | >2.50 | 1.25 | >2.50 | 1.25 |
| *H. odoratissimum* | L | 2.50 | 2.50 | 2.50 | 2.50 | 2.50 | 2.50 | 2.50 | 2.50 | 0.63 | 0.31 | 2.50 | 0.31 | >2.50 | 0.31 | >2.50 | 0.63 |
| *G. corymbosum* | L | 2.50 | 2.50 | 2.50 | 2.50 | 2.50 | 2.50 | 2.50 | 1.25 | 0.63 | 0.63 | >2.50 | 0.31 | 0.63 | 0.63 | 1.25 | 1.25 |
| *A. annua* | L | 1.25 | 1.25 | 1.25 | 1.25 | 1.25 | 1.25 | 1.25 | 1.25 | 0.63 | 0.63 | >2.50 | 0.31 | >2.50 | 1.25 | 1.25 | 1.25 |
| *A. afra* | L | 1.25 | 1.25 | 1.25 | 1.25 | 1.25 | 1.25 | 1.25 | 1.25 | 0.63 | 0.63 | >2.50 | 0.63 | 1.25 | 1.25 | 1.25 | 1.25 |

MIC – minimal inhibition concentration and MQSIC – minimal quorum sense inhibition concentration. *n* = 1.

Supplementary Files F1 to F5 used for the tentative annotation of compounds from *Terminalia phanerophlebia* using MassLynx.


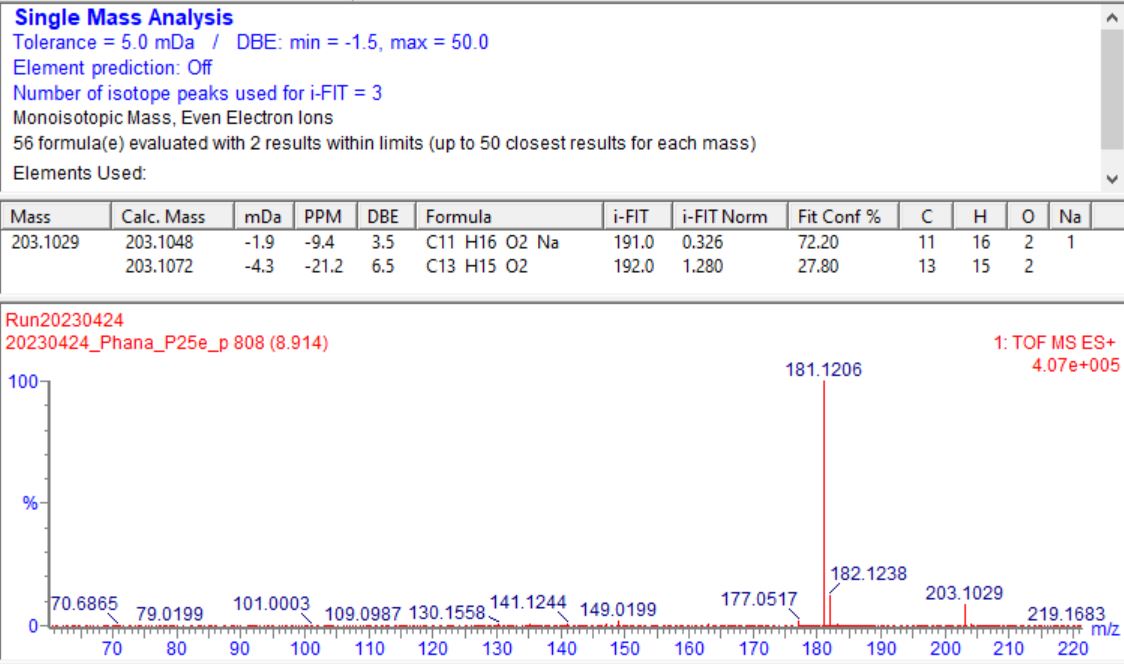


[M+Na]^+^

Figure F1: Annotation for compound 1 on MassLynx


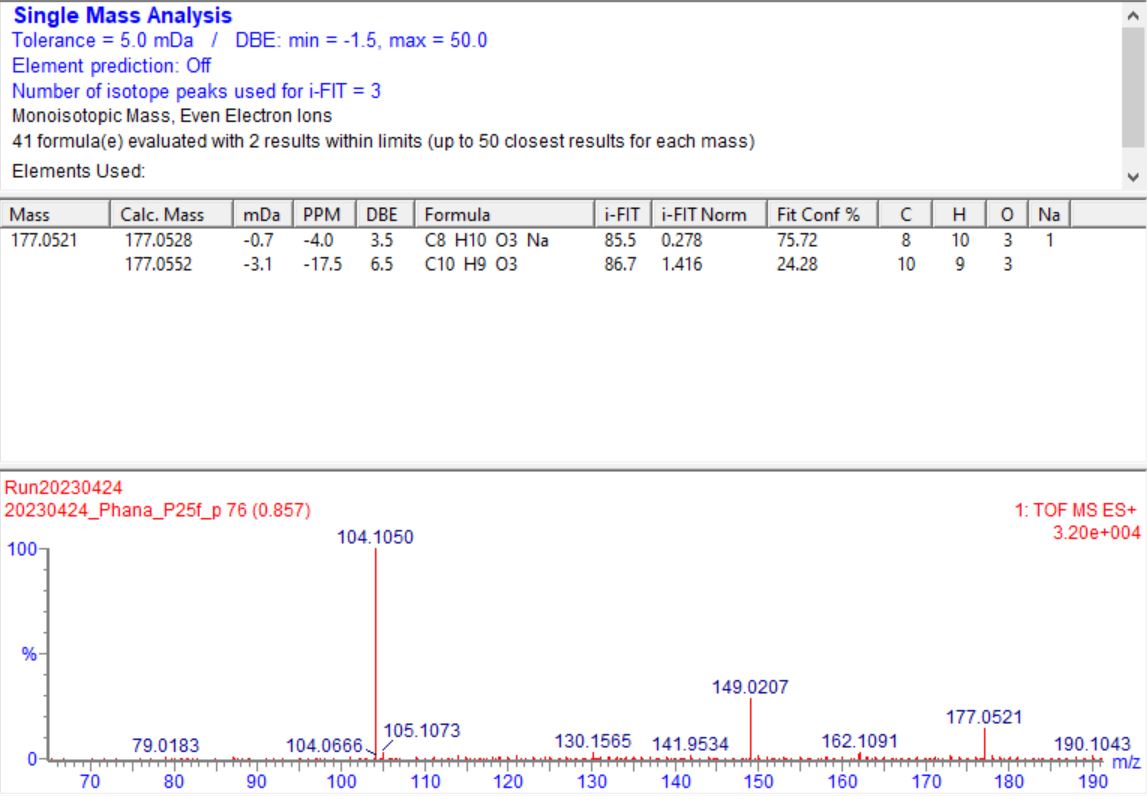


[M+Na]^+^

Figure F2: Annotation for compound **2** on MassLynx


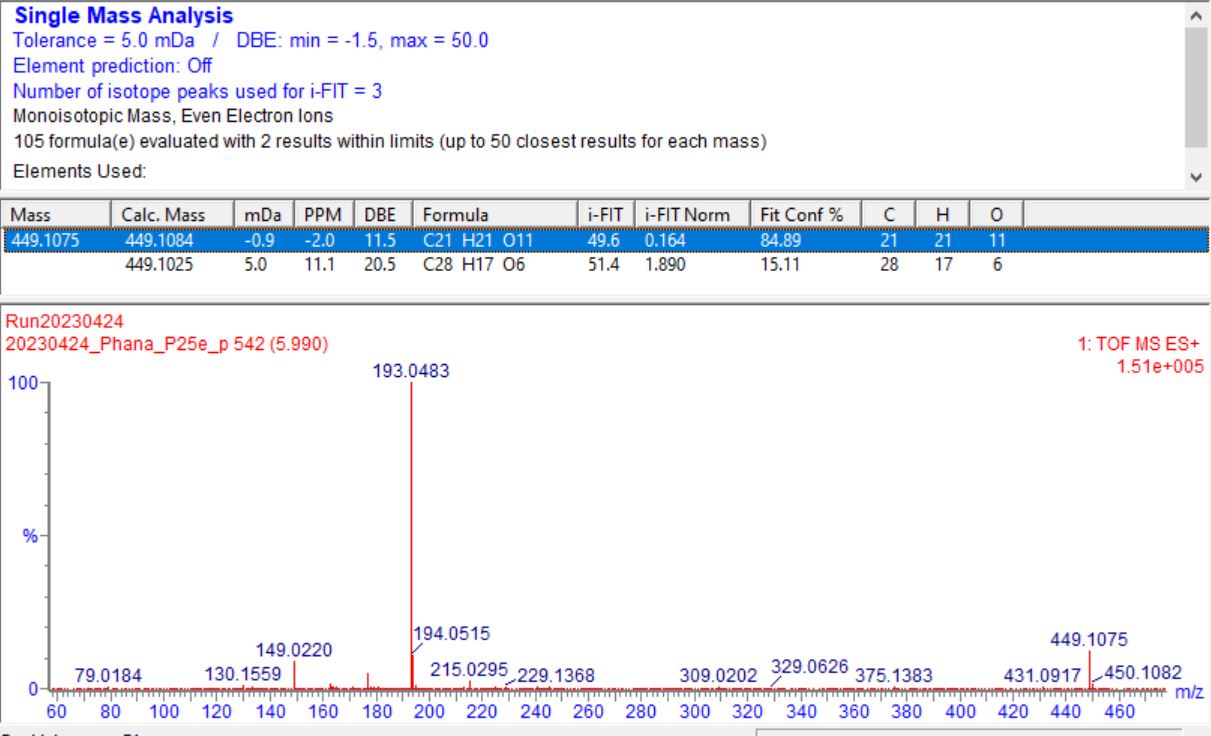


[M+H]^+^

Figure F3: Annotation for compound **3** on MassLynx


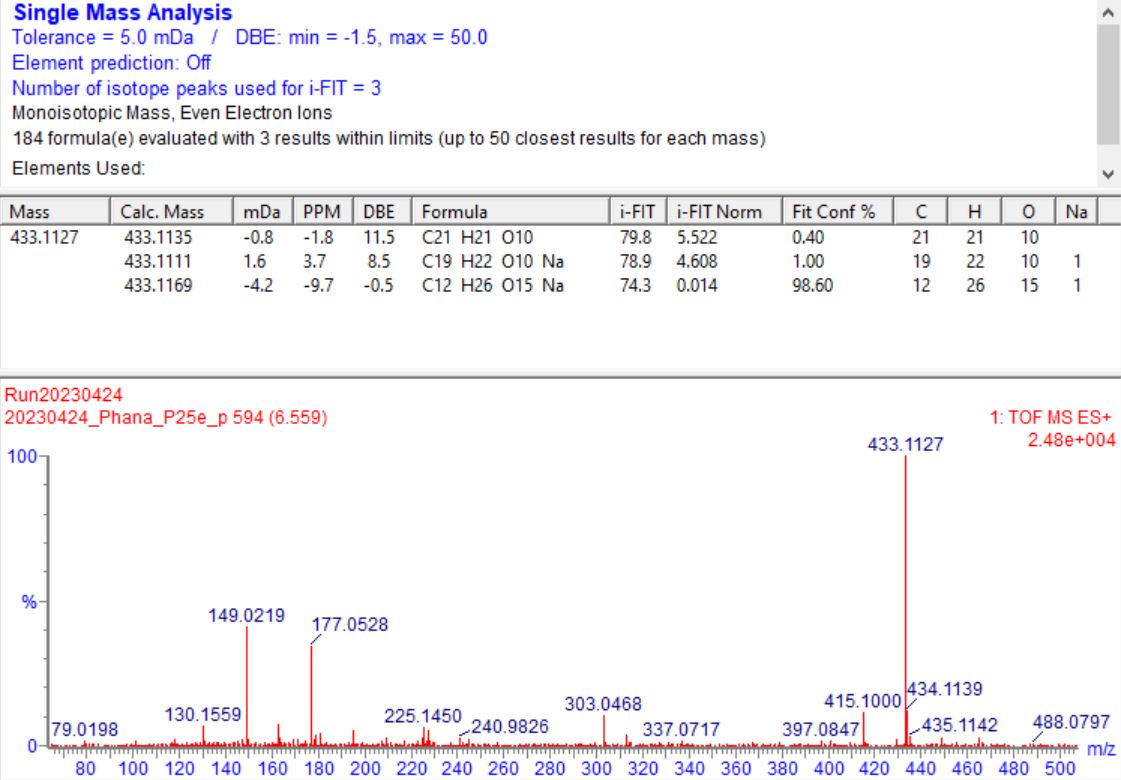
Figure F4: Annotation for compound **4** on MassLynx

[M+H]^+^


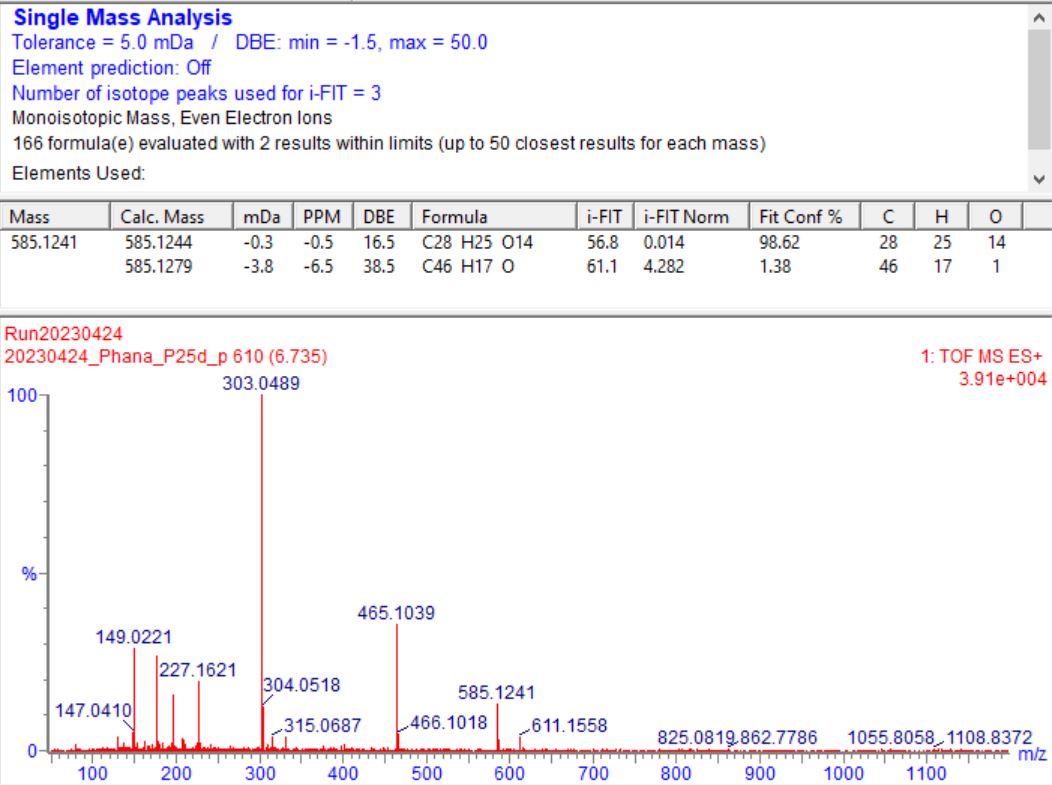
Figure F5: Annotation for compound **5** on MassLynx

[M+H]^+^

[M+H]^+^

.

**References**

1. Hutchings A: *Zulu medicinal plants: An inventory.* University of Natal press; 1996.

2. Ramabulana A-T, Petras D, Madala NE, Tugizimana F: **Metabolomics and molecular networking to characterize the chemical space of four Momordica plant species.** *Metabolites* 2021, **11:**763.

3. Muronga M, Quispe C, Tshikhudo PP, Msagati TAM, Mudau FN, Martorell M, Salehi B, Abdull Razis AF, Sunusi U, Kamal RM, Sharifi-Rad J: **Three Selected Edible Crops of the Genus Momordica as Potential Sources of Phytochemicals: Biochemical, Nutritional, and Medicinal Values.** *Frontiers in Pharmacology* 2021, **12**.

4. Amoo SO, Ndhlala AR, Finnie JF, Van Staden J: **Antibacterial, antifungal and anti-inflammatory properties of Burchellia bubalina.** *South African Journal of Botany* 2009, **75:**60-63.

5. Mhlongo LS, Van Wyk BE: **Zulu medicinal ethnobotany: new records from the Amandawe area of KwaZulu-Natal, South Africa.** *South African Journal of Botany* 2019, **122:**266-290.

6. Rana M, Rana M, Sharma D, Chauhan P: **Commonly used medicinal plants in tehsil Bangana, district una, Himachal Pradesh.** *Journal of Ayurvedic and Herbal Medicine* 2017, **3:**102-107.

7. Kambizi L: **Indigenous plants for ethnoveterinary uses in the Pondoland, South Africa.** In *XXIX International Horticultural Congress on Horticulture: Sustaining Lives, Livelihoods and Landscapes (IHC2014): V World 1125*2014: 309-314.

8. Semenya SS, Maroyi A: **Ethnobotanical survey of plants used by Bapedi traditional healers to treat tuberculosis and its opportunistic infections in the Limpopo Province, South Africa.** *South African Journal of Botany* 2019, **122:**401-421.

9. Ye S, Hu J, Liu Z, Liang M: **Progress and Research Trends on Catha edulis (Vahl) Endl. (Catha edulis): A Review and Bibliometric Analysis.** *Frontiers in Pharmacology* 2021, **12**.

10. Grace O, Prendergast H, Jäger A, Van Staden J, Van Wyk A: **Bark medicines used in traditional healthcare in KwaZulu-Natal, South Africa: An inventory.** *South African Journal of Botany* 2003, **69:**301-363.

11. Rojas-Sandoval J: **Hedychium gardnerianum (kahili ginger).** 2023.

12. Wu Z, Raven P, Hong D: **Flora of China; Missouri Botanical Garden Press: St.** *Louis, MO, USA* 1994, **2013**.

13. Adebayo SA, Amoo SO, Mokgehle SN, Aremu AO: **Ethnomedicinal uses, biological activities, phytochemistry and conservation of African ginger (Siphonochilus aethiopicus): A commercially important and endangered medicinal plant.** *J Ethnopharmacol* 2021, **266:**113459.

14. Seile BP, Bareetseng S, Koitsiwe MT, Aremu AO: **Indigenous Knowledge on the Uses, Sustainability and Conservation of African Ginger (Siphonochilus aethiopicus) among Two Communities in Mpumalanga Province, South Africa.** *Diversity* 2022, **14:**192.

15. Nsuala BN, Enslin G, Viljoen A: **“Wild cannabis”: A review of the traditional use and phytochemistry of Leonotis leonurus.** *Journal of Ethnopharmacology* 2015, **174:**520-539.

16. Ezema CA, Aguchem RN, Aham EC, Ezeorba WFC, Okagu IU, Ezeorba TPC: **Salvia africana-lutea L.: a review of ethnobotany, phytochemistry, pharmacology applications and future prospects.** *Advances in Traditional Medicine* 2023.

17. Kamatou GPP, Makunga NP, Ramogola WPN, Viljoen AM: **South African Salvia species: A review of biological activities and phytochemistry.** *Journal of Ethnopharmacology* 2008, **119:**664-672.

18. Rattray RD, Van Wyk B-E: **The Botanical, Chemical and Ethnobotanical Diversity of Southern African Lamiaceae.** *Molecules* 2021, **26:**3712.

19. Tietjen I, Gatonye T, Ngwenya BN, Namushe A, Simonambanga S, Muzila M, Mwimanzi P, Xiao J, Fedida D, Brumme ZL, et al: **Croton megalobotrys Müll Arg. and Vitex doniana (Sweet): Traditional medicinal plants in a three-step treatment regimen that inhibit in vitro replication of HIV-1.** *Journal of Ethnopharmacology* 2016, **191:**331-340.

20. Mathibela MK, Potgieter MJ, Tshikalange TE: **Medicinal plants used to manage sexually transmitted infections by Bapedi traditional health practitioners in the Blouberg area, South Africa.** *South African Journal of Botany* 2019, **122:**385-390.

21. Demgne OMF, Damen F, Fankam AG, Guefack MF, Wamba BEN, Nayim P, Mbaveng AT, Bitchagno GTM, Tapondjou LA, Penlap VB, et al: **Botanicals and phytochemicals from the bark of Hypericum roeperianum (Hypericaceae) had strong antibacterial activity and showed synergistic effects with antibiotics against multidrug-resistant bacteria expressing active efflux pumps.** *J Ethnopharmacol* 2021, **277:**114257.

22. Kumari A, Baskaran P, Van Staden J: **In vitro propagation via organogenesis and embryogenesis of Cyrtanthus mackenii: a valuable threatened medicinal plant.** *Plant Cell, Tissue and Organ Culture (PCTOC)* 2017, **131:**407-415.

23. Ramadwa TE, Dzoyem JP, Adebayo SA, Eloff JN: **Ptaeroxylon obliquum leaf extracts, fractions and isolated compounds as potential inhibitors of 15-lipoxygenase and lipopolysaccharide-induced nitric oxide production in RAW 264.7 macrophage cells.** *South African Journal of Botany* 2022, **147:**192-196.

24. Irungu BN, Orwa JA, Gruhonjic A, Fitzpatrick PA, Landberg G, Kimani F, Midiwo J, Erdélyi M, Yenesew A: **Constituents of the Roots and Leaves of Ekebergia capensis and Their Potential Antiplasmodial and Cytotoxic Activities.** *Molecules* 2014, **19:**14235-14246.

25. Komane BM, Olivier EI, Viljoen AM: **Trichilia emetica (Meliaceae) – A review of traditional uses, biological activities and phytochemistry.** *Phytochemistry Letters* 2011, **4:**1-9.

26. Semenya S, Potgieter M, Tshisikhawe M: **Use, conservation and present availability status of ethnomedicinal plants of Matebele-Village in the Limpopo Province, South Africa.** *African Journal of Biotechnology* 2013, **12**.

27. Khalil N, Elhady SS, Diri RM, Fekry MI, Bishr M, Salama O, El-Zalabani SM: **Salicylic Acid Spraying Affects Secondary Metabolites and Radical Scavenging Capacity of Drought-Stressed Eriocephalus africanus L.** *Agronomy* 2022, **12:**2278.

28. Serabele K, Chen W, Combrinck S: **Chapter 10 - Helichrysum odoratissimum.** In *The South African Herbal Pharmacopoeia.* Edited by Viljoen A, Sandasi M, Fouche G, Combrinck S, Vermaak I: Academic Press; 2023: 247-258

29. Kizito IG, Mohammed K: **Phytochemical analysis, antimicrobial and antioxidant activities of leaf extract of Vernonia tigna Klatt (Asteraceae).** *World* 2022, **3:**016-023.

30. Hien TT, White NJ: **Qinhaosu.** *The Lancet* 1993, **341:**603-608.

31. Liu NQ, Van der Kooy F, Verpoorte R: **Artemisia afra: A potential flagship for African medicinal plants?** *South African Journal of Botany* 2009, **75:**185-195.
